# Divergent *C. elegans* toxin alleles are suppressed by distinct mechanisms

**DOI:** 10.7554/eLife.106269

**Published:** 2026-08-11

**Authors:** Stefan Zdraljevic, Laura Walter-McNeill, Giancarlo N Bruni, Joshua S Bloom, JB Collins, Daniel HW Leighton, Heriberto Marquez, Noah Alexander, Leonid Kruglyak

**Affiliations:** 1 Department of Human Genetics, University of California, Los Angeles Los Angeles United States; 2 Department of Biological Chemistry, University of California, Los Angeles Los Angeles United States; 3 Howard Hughes Medical Institute Chevy Chase United States; 4 Department of Biology, Johns Hopkins University Baltimore United States; University at Buffalo, State University of New York United States; Max Planck Institute for Biology Tübingen Germany

**Keywords:** toxin-antidote, self element, gene drive, hyper-divergent, *C. elegans*

## Abstract

Toxin-antidote elements (TAs) are selfish DNA sequences that bias their transmission to the next generation. TAs typically consist of two linked genes: a toxin and an antidote. The toxin kills progeny that do not inherit the TA, while the antidote counteracts the toxin in progeny that inherit the TA. We previously discovered two TAs in *Caenorhabditis elegans* that follow the canonical TA model of two linked genes: *peel-1/zeel-1* and *sup-35/pha-1*. Here, we report a new TA that exists in three distinct states across the *C. elegans* population. The canonical TA, which is found in isolates from the Hawaiian Islands, consists of two genes that encode a maternally deposited toxin (TMRL-1) and a zygotically expressed antidote (AMRL-1). The toxin induces larval lethality in embryos that do not inherit the antidote gene. A second version of the TA has lost the toxin gene but retains a partially functional antidote. Most *C. elegans* isolates, including the standard laboratory strain N2, carry a highly divergent allele of the toxin that has retained its activity, but have lost the antidote through pseudogenization. Multiple lines of evidence suggest that the N2 *tmrl-1* allele is likely recognized by piRNAs, leading to MUT-16-dependent 22G small interfering RNA (siRNA) production and post-transcriptional silencing of the transcript. The N2 haplotype represents the first naturally occurring unlinked toxin-antidote system where the toxin is post-transcriptionally suppressed by endogenous small RNA pathways.

## Introduction

Toxin-antitoxin or toxin-antidote (TA) elements are extreme examples of selfish genetic elements that typically consist of two linked genes encoding a toxin and a cognate antidote. The toxin kills individuals that don’t inherit the element and hence lack the antidote to counteract the effects of the toxin (Beeman et al., 1992; Ben-David et al., 2017; Seidel et al., 2008; Ben-David et al., 2021b; Jurėnas et al., 2022). TA elements are ubiquitous in bacteria and have been shown to function as defense mechanisms against bacteriophages, either by directly inhibiting the infection cycle of a phage or by targeting host factors to prevent the spread of mature virions (LeRoux and Laub, 2022). Like other immune genes involved in pathogen recognition, TA components are poorly conserved across bacteria because they evolve rapidly to maintain a competitive edge against their target phages (Shultz and Sackton, 2019; Daugherty and Malik, 2012).

While the presence of extremely toxic genes in bacteria can be explained by their role in phage-defense systems, the maintenance of TA elements in metazoans is more mysterious. TA elements are common in hermaphroditic *Caenorhabditis* nematodes (Ben-David et al., 2017; Seidel et al., 2008; Ben-David et al., 2021b; Seidel et al., 2011; Noble et al., 2021), an observation consistent with recent analytical results that selfing can promote the spread of TA elements (Rockman, 2024; Wang et al., 2024). Each of the known *Caenorhabditis* TA elements resides in a hyper-variable genomic region, which suggests that these elements predate the evolution of selfing (Lee et al., 2021) or have contributed to the suppression of gene flow between hyper-variable haplotypes (Rockman, 2024). TA elements are expected to drive to fixation in outcrossing populations. Once it is fixed or nearly fixed, it loses its selective advantage, and there is no selective pressure to maintain it. Therefore, unless a fixed TA provides an additional fitness advantage, the element will likely degrade over time. A recent report suggests that *peel-1*, the toxin component of the first TA element discovered in *Caenorhabditis elegans*, increases host fitness in laboratory conditions, raising the possibility that toxic genes can take on new roles that allow them to be maintained at high frequencies in primarily selfing nematode populations (Long et al., 2023).

Here, we describe a novel maternally inherited TA element in *C. elegans* with distinctive features. The maternally deposited toxin causes larval arrest rather than embryonic lethality, raising the question of how the toxicity is delayed to this late developmental stage. At the population level, we identified three clades with distinct haplotypes at the new TA locus, the most common of which appears to possess a functional toxin without an antidote. We show that the toxin in this haplotype is likely recognized by endogenous piRNA machinery for perpetual silencing by the 22G small RNA (sRNA) pathway. Thus, a vast majority of *C. elegans* strains harbor an unlinked TA system that has no ability to act as a gene drive.

## Results

### Identification of a novel *C. elegans* TA element

**Figure 1.**
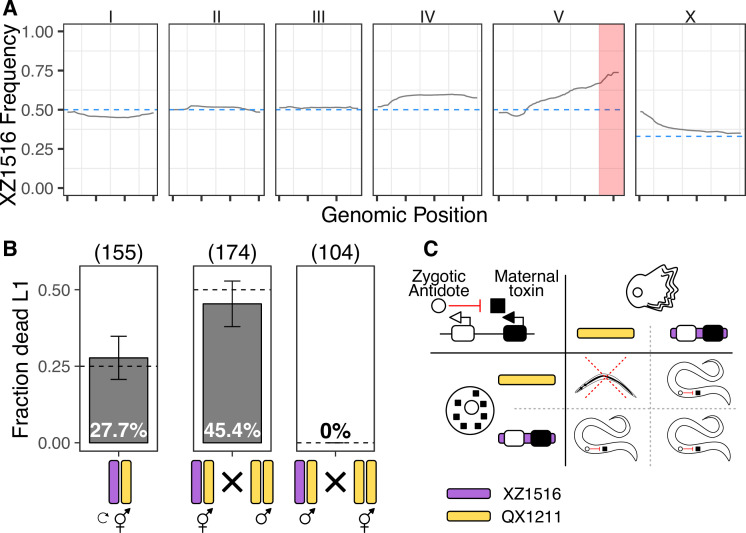
Discovery and characterization of a novel toxin-antidote (TA). (**A**) The gray line represents the frequency of XZ1516 alleles across the genome after four generations of intercrossing with QX1211. Each panel corresponds to a *C. elegans* chromosome and each x-axis tick indicates 5 Mb. The dotted blue line represents the expected allele frequency for each chromosome with no selection. The region highlighted in red on the right side of chromosome V shows the greatest allele frequency deviation from expectation. (**B**) Crosses between XZ1516 (purple) and QX1211 (yellow) establish the inheritance pattern of the TA element. Bar plots show the fraction of dead L1s observed in each cross. Error bars indicate 95% binomial confidence intervals calculated using the normal approximation method. Crosses from left to right: selfing of XZ1516/QX1211 heterozygous hermaphrodites; XZ1516/QX1211 heterozygous hermaphrodites crossed to QX1211 males; XZ1516/QX1211 heterozygous males crossed to QX1211 hermaphrodites. The observed fraction of dead L1s was not significantly different from the expected fractions for a maternally inherited TA element, exact binomial test. (**C**) Model of the TA inheritance. Punnett square shows the lethality pattern expected in progeny from selfing of XZ1516/QX1211 heterozygous hermaphrodites. A maternally deposited toxin (black square) is present in all progeny and causes L1 lethality unless a zygotically expressed antidote (white circle) is also present.

To study the phenotypic effects of genetic variation in *C. elegans*, we generated large cross populations between highly divergent strains—XZ1516 × QX1211 and XZ1516 × DL238. We chose these strains because they are compatible at the two incompatibility loci we previously discovered: *peel-1/zeel-1* and *sup-35/pha-1* (Ben-David et al., 2017; Seidel et al., 2008). We introduced a *fog-2* loss-of-function allele, which feminizes hermaphrodites and prevents them from selfing, into each genetic background to facilitate the construction of large cross populations and intercrossed each population for 10 generations, with minimal selection. Despite minimal selection across each generation, whole-genome sequencing across generations revealed multiple genomic loci with allele frequency distortions, indicating that genetic differences at these loci influenced relative fitness in standard laboratory growth conditions (Figure 1—figure supplement 1A). We observed that by generation four of the XZ1516 × QX1211 cross, the XZ1516 allele frequency rose to 75% on the right arm of chromosome V (Figure 1A). We also observed allele frequency distortion at this region in later generations of the XZ1516 × DL238 cross, which suggested that the same underlying genetic difference was being selected in both crosses. Based on previous studies, we hypothesized that this strong depletion of the QX1211 genotype by generation four is caused by a TA element at this locus (Burga et al., 2019). To test this hypothesis, we performed new crosses between QX1211 and XZ1516 and tracked the phenotypes and genotypes of F2 progeny. We observed that ~27% of the F2 self-progeny of heterozygous QX1211/XZ1516 F1 hermaphrodites arrested as L1 larvae (Figure 1B). The observed larval arrest phenotype is reminiscent of the rod-like larval lethal (*rod*) phenotype (Figure 1—figure supplement 1B; Rocheleau et al., 2002). When we crossed QX1211/XZ1516 F1 hermaphrodites to QX1211 males, ~45% of the progeny exhibited the *rod* phenotype, while we observed no *rod* progeny in the reciprocal cross between QX1211/XZ1516 F1 males and QX1211 hermaphrodites (Figure 1B). We used PCR genotyping to verify that all *rod* progeny were homozygous for QX1211 alleles at the locus on the right arm of chromosome V that displayed the allele frequency distortion in the mapping populations. This inheritance pattern suggests that the XZ1516 genome encodes a maternally inherited toxin and a linked zygotically expressed antidote that form a novel TA element responsible for the observed allele frequency distortions on the right arm of chromosome V (Figure 1C). We observed the same F2 phenotypes in crosses between DL238 and XZ1516, indicating that DL238 is a noncarrier of the TA element (Figure 1—figure supplement 1C).

**Figure 1—figure supplement 1.**
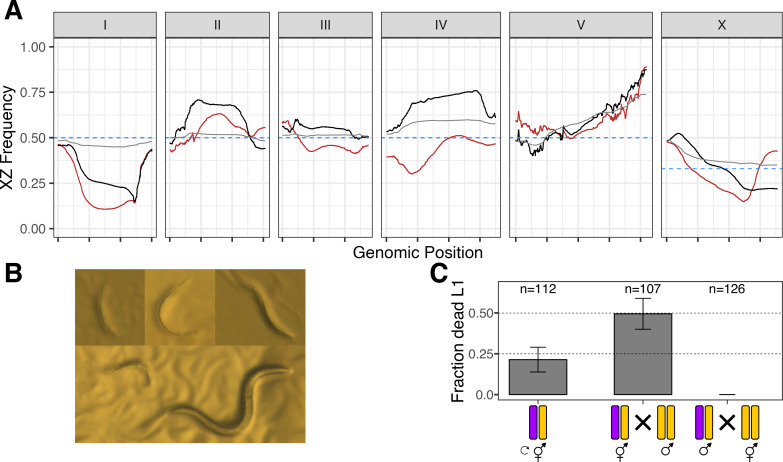
DL238 is also susceptible to the XZ1516 toxin-antidote (TA). (**A**) Estimates of the XZ1516 allele frequency at each position in the genome after four (gray) or ten generations of intercrossing with QX1211 (black, gray) or DL238 (red). Each panel corresponds to a *C. elegans* chromosome and each x-axis tick represents 5 Mb. The dotted blue line represents the expected allele frequency for each chromosome. (**B**) Top three panels are representative images of *rod*-like lethality phenotype caused by the *tmrl-1* toxin. The bottom panel is a *rod* worm next to a normally developing worm derived from the same cross. (**C**) Crosses between XZ1516 (purple) and DL238 (yellow) that establish the inheritance pattern of the TA element. The y-axis represents the fraction of dead L1s observed for each cross depicted on the x-axis. Crosses from left to right: selfing of XZ1516/DL238 heterozygous; XZ1516/DL238 heterozygous hermaphrodites crossed to DL238 males; XZ1516/DL238 heterozygous males crossed to DL238 hermaphrodites. The observed fraction of dead L1s was not significantly different from the expected fractions for a maternally inherited TA element, exact binomial test.

### Identifying the components of the XZ1516 TA element

**Figure 2.**
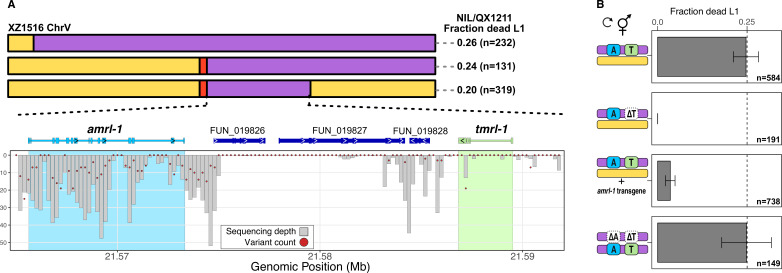
Identification of the toxin-antidote (TA) components. (**A**) Localization of the TA element genes in XZ1516. Top panel: Strain genotypes of near-isogenic lines (NILs) are displayed as colored rectangles (XZ1516 in purple; QX1211 in yellow; Cas9-induced deletion in red) for chromosome V. The fraction of L1 lethality after selfing of the NIL/QX1211 hermaphrodites is shown to the right of each NIL. The bottom panel depicts a summary of QX1211 sequencing reads aligned to the XZ1516 genome corresponding to the mapped TA element. Gray bars denote short-read sequencing depth in 200 bp windows, and red dots denote the number of variants detected between QX1211 and XZ1516 in each window. The XZ1516 and QX1211 genomes are so diverged that short reads derived from QX1211 don’t align to the XZ1516 genome in the 200 bp windows with no corresponding read depth, as indicated by a lack of a gray bar. The toxin and antidote genes are highlighted in green and light blue, respectively. (**B**) Knockout and transgenic rescue experiments define the TA components. Bar plots denote the fraction of dead L1s derived from selfing F1 heterozygous individuals. Error bars indicate 95% binomial confidence intervals calculated using the normal approximation method. Blue and green boxes with ‘A’ and ‘T’ indicate intact antidote and toxin genes, respectively; white boxes indicate deletions of these genes. XZ1516 genotypes are depicted in purple and QX1211 genotypes are depicted in yellow. Panels from top to bottom: XZ1516/QX1211 control cross, the observed lethality is not significantly different from the expected 25%; toxin knockout cross to QX1211, the observed lethality is significantly different from the expected 25% p=1.38e-31; antidote transgenic rescue cross the observed lethality is significantly different from the expected 25% p=1.22e-53; toxin and antidote double knockout cross to XZ1516, the observed lethality is not significantly different from the expected 25%. An exact binomial test was used to determine significance.

To isolate the XZ1516 TA element, we introgressed the right arm of chromosome V from XZ1516 into QX1211. We confirmed the identity of the resulting near-isogenic line (NIL) by whole-genome sequencing and verified the presence of the TA element with crosses (Figure 2A). We were unable to further localize the TA location with standard fine-mapping approaches, likely because of low recombination rates near the ends of *C. elegans* chromosomes (Rockman and Kruglyak, 2009). To overcome the limited natural recombination in this region, we developed a method to induce targeted recombination at double-stranded DNA breaks generated by Cas9 (Zdraljevic et al., 2023). This approach enabled us to localize the TA element to a 50 kb region containing 10 candidate genes. We tested these genes for potential toxin or antidote activity by systematically knocking them out in the XZ1516 genetic background (Figure 2A).

**Figure 2—figure supplement 1.**
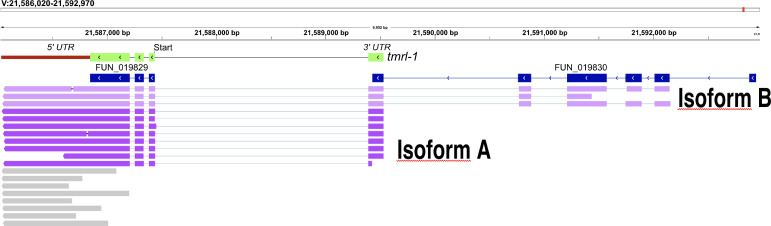
Isoforms of the *tmrl-1* toxin. Long-read RNA sequencing reads of the different *tmrl-1* isoforms aligned to the XZ1516 genome visualized in the Integrative Genomics Viewer (IGV). Light purple reads represent isoform B, the dark purple reads represent isoform A, and gray reads represent the 5’ UTR of *tmrl-1*. The functional gene model of *tmrl-1* is depicted in green above the computationally predicted ‘FUN’ gene models.

**Figure 2—figure supplement 2.**
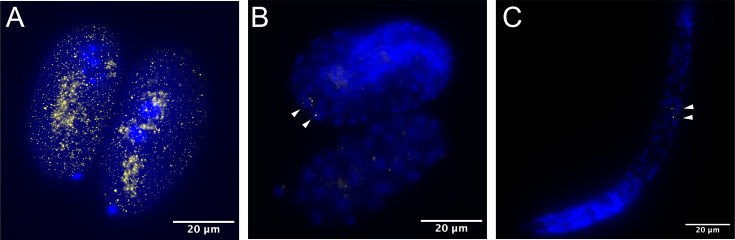
The tmrl-1 transcript persists until the L1 stage. Representative *tmrl-1* FISH images in XZ1516. Yellow corresponds to *tmrl-1* transcript puncta, and nuclei staining with DAPI is in blue. (**A**) Max projection of one- and two-cell embryos. (**B**) Post-gastrulation embryos showing *tmrl-1* localization to the Z2/Z3 cells. (**C**) L1 larvae showing *tmrl-1* localization to the Z2/Z3 cells.

We isolated three deletion strains that did not induce larval lethality when crossed to QX1211, suggesting that these strains lacked the toxin (Figure 2B). The computationally predicted gene FUN_019829 is deleted in all three of these strains, and in one of the strains, only this gene is deleted, confirming that this gene encodes the toxin. We hereafter refer to FUN_019829 as *tmrl-1* (Toxin-induced Maternal Rod Lethal). We were unable to generate homozygous deletion lines of gene FUN_019825, which suggested that this gene is either essential for survival or encodes the antidote. We successfully isolated homozygous deletion lines of FUN_019825 in a Δ*tmrl-1* genetic background, indicating that this gene encodes the antidote. We hereafter refer to FUN_019825 as *amrl-1* (Antidote of Maternal Rod Lethal). We showed that a strain with deletions of both *tmrl-1* and *amrl-1* phenocopies susceptible strains in crosses (Figure 2B).

To determine whether *amrl-1* is sufficient to suppress *tmrl-1*-induced larval lethality, we constructed a rescue plasmid to drive *amrl-1* expression with a constitutive promoter. We injected the rescue plasmid into XZ1516, crossed individuals harboring the rescue array to a TA-susceptible strain, and selfed the F1 progeny that inherited the array. We observed a dramatic reduction in larval arrest from 25% to 3.5% in F2 progeny, and all F2 progeny that inherited the rescue array survived. These results confirm that *amrl-1* is sufficient to suppress *tmrl-1* toxicity (Figure 2B).

Long-read RNA sequencing revealed two distinct *tmrl-1* isoforms, a short isoform with three predicted exons and a long isoform with eight predicted exons (Figure 2—figure supplement 1). We constructed plasmids with inducible versions of each *tmrl-1* isoform. When we injected susceptible strains with the short *tmrl-1* isoform array, every F1 individual carrying the array died, with 64% of larvae exhibiting the *rod* phenotype, indicating that uninduced expression levels of the short *tmrl-1* isoform are sufficient to induce lethality. By contrast, we were able to isolate susceptible strains that maintained the long *tmrl-1* isoform array or a short *tmrl-1* isoform array with a premature stop codon in *tmrl-1*. We observed no *rod* progeny upon induction of these arrays, indicating that the short isoform encodes the functional toxin, and that the toxin acts as a protein.

Because lethality only occurs at the L1 stage, we reasoned that *tmrl-1* might be deposited in embryos as a transcript and sequestered from translation. We performed fluorescence in situ hybridization (FISH) on developing XZ1516 embryos and larvae with RNA probes that target the *tmrl-1* mRNA. We observed *tmrl-1* puncta as early as the two-cell embryo stage (Figure 2—figure supplement 2A), indicating that *tmrl-1* transcripts are maternally deposited because zygotic transcription does not initiate prior to the four-cell stage (Robertson and Lin, 2015). At later embryonic and L1 development stages, the *tmrl-1* transcript is localized to two cells that likely correspond to the primordial germ cells (Figure 2—figure supplement 2B–C).

### Genomic and population features of the *tmrl-1*/*amrl-1* TA element

**Figure 3.**
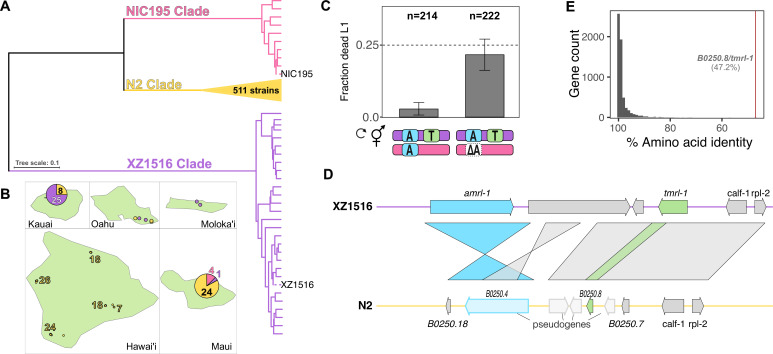
Demographics of the *tmrl-1/amrl-1* toxin-antidote (TA). (**A**) A dendrogram showing the relatedness of 550 wild *C. elegans* strains at the TA locus. Branches are colored to represent the three distinct clades, where purple denotes the XZ1516-like clade, yellow denotes the N2-like clade, and pink denotes the NIC195-like clade. (**B**) Isolation location of strains collected in Hawaii. Pie charts show the number of isolates from each clade when multiple strains were collected at one location, with colors as in A. (**C**) Bar plots show the fraction of dead L1s in crosses between XZ1516 and NIC195 (left) and between XZ1516 and NIC195 with its antidote allele knocked out (right), indicating that this antidote is active against the XZ1516 toxin. Error bars indicate 95% binomial confidence intervals calculated using the normal approximation method. The observed lethality in the NIC195 × XZ1516 cross is significantly different from the expected 25% (p=6.14e-19, exact binomial test), while the antidote knockout difference is not significantly different. (**D**) Synteny plot of the TA region between the XZ1516 (top) and N2 (bottom) genomes. The TA components *tmrl-1* and *amrl-1* are colored green and blue, respectively. (**E**) Percent amino acid identity of ~5500 one-to-one orthologs identified between the XZ1516 and N2 genomes. Amino acid identity for *tmrl-1* is indicated with a red line.

The XZ1516 genomic region surrounding the *tmrl-1*/*amrl-1* TA element is hyper-divergent from the reference (N2) genome (Lee et al., 2021). We characterized the genetic variation at this region in the *C. elegans* population by calculating the relatedness of 550 wild isolates (Cook et al., 2017). This analysis separated the population into three distinct clades: an XZ1516-like TA clade, which contains 29 strains, a 10-strain clade, and an N2-like susceptible clade composed of 511 strains, including QX1211 and DL238 (Figure 3A). We verified that the 28 additional isolates with the XZ1516-like haplotype have intact *tmrl-1/amrl-1* genes by aligning sequencing reads from these isolates to the XZ1516 genome assembly. All but four of the isolates in the XZ1516-like clade were collected within three miles of each other on the island of Kauai, two were isolated on Oahu, and one each on Maui and Moloka’i. Four of the isolates from the 10-strain clade were isolated on Maui, and the remaining six are globally distributed, while strains with the susceptible haplotype, which represent the majority of the known *C. elegans* isolates, are globally distributed and present on all the Hawaiian islands with the exception of Moloka’i (Figure 3B).

**Figure 3—figure supplement 1.**
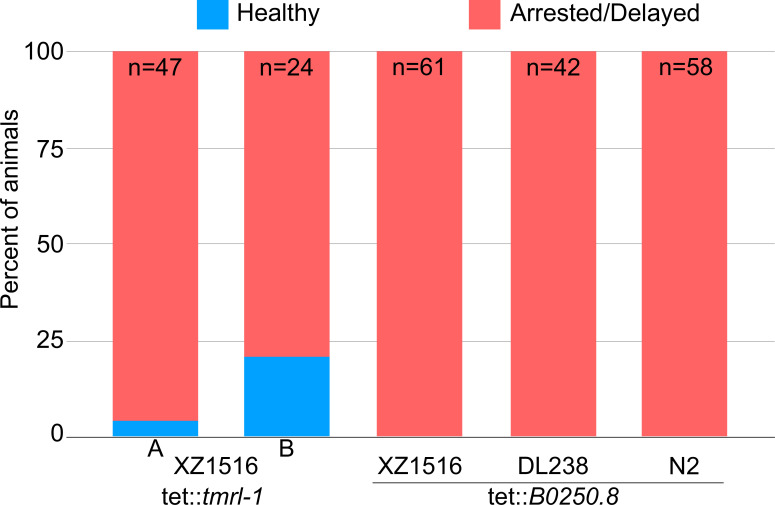
Phenotypes induced by the two *tmrl-1* alleles. The percent of healthy (blue) or arrested or delayed (red) individuals is shown on the y-axis. The first two bars represent technical replicates of tetracycline-induced *tmrl-1* in the XZ1516 genetic background. The remaining three bars correspond to tetracycline-induced *B0250.8* in XZ1516, DL238, and N2, respectively.

The 10-strain clade carries a haplotype that does not contain a gene resembling the toxin. However, this haplotype does carry a divergent *amrl-1* allele that is predicted to contain a full-length coding sequence. We therefore asked whether this *amrl-1* allele is capable of suppressing the toxic effects of *tmrl-1*. We observed the *rod* phenotype in only 3% of F2 progeny derived from crosses between XZ1516 and a representative strain with this haplotype, NIC195 (Figure 3C), indicating that this antidote is at least partially functional. When we knocked out the *amrl-1* allele in NIC195, 22.5% of F2 progeny were *rod*, confirming that this divergent allele confers reduced susceptibility to the effects of *tmrl-1* (Figure 3C).

While the previously described *C. elegans* TA elements are characterized by their absence in susceptible strains (Ben-David et al., 2017; Seidel et al., 2008), all members of the N2-like susceptible clade harbor a divergent allele of *tmrl-1* with an intact coding sequence, as well as a pseudogenized version of *amrl-1*. The *tmrl-1/amrl-1* genomic region contains several genomic rearrangements between XZ1516 and N2, including likely inversion events that occurred between *amrl-1* and its corresponding divergent N2 allele, *B0250.4*; these inversions may have contributed to its pseudogenization (Figure 3D). While synteny is maintained between *tmrl-1* and the corresponding divergent N2 allele, *B0250.8*, many of the surrounding N2 genes are predicted to be pseudogenized. The divergence between *tmrl-1* and *B0250.8* is the highest among one-to-one orthologs in the XZ1516 and N2 genomes (nucleotide identity: 63%; protein identity: 47%) (Figure 3E). We estimated the divergence time for these two alleles under the assumption of neutrality to be between 160 and 325 million generations based on the estimates of divergence at synonymous sites (dS) (Gillespie and Langley, 1979; Thomas et al., 2015). This implausibly old estimate suggests that positive selection has been driving the diversification of this gene. The fact that *B0250.8* has an intact coding sequence raises the question of whether this gene has maintained its function as a toxin, and if so, how individuals with this haplotype can exist without a functional antidote.

To determine whether *B0250.8* acts as a toxin, we used a tetracycline-inducible system to drive the expression of *B0250.8* in XZ1516, DL238, and N2. We hatched worms carrying the inducible array on doxycycline plates to induce *B0250.8* expression and recorded their phenotypes 48 hr after hatching. All worms expressing *B0250.8* displayed a variety of abnormal phenotypes (N2 [n=58]; DL238 [n=42]; XZ1516 [n=61]), which are likely caused by induced expression of *B0250.8* in a wide range of tissue types. Notably, we observed the stereotypical *tmrl-1*-dependent *rod* phenotype at low frequencies in all strains (2/58 N2, 5/42 DL238, 4/61 XZ1516). Furthermore, the abnormal phenotypes we observed upon induction of *B0250.8* were also seen upon induction of *tmrl-1* (Figure 3—figure supplement 1; Supplementary file 7), which suggests that *B0250.8* (hereafter N2 *tmrl-1*) has retained its function as a toxin. The presence of a functional toxin and a pseudogenized antidote in N2-like strains suggests that a different mechanism suppresses the toxicity associated with the N2 *tmrl-1* and that this suppression mechanism does not affect the XZ1516 *tmrl-1* toxin (Figure 1B).

### Small-RNA-mediated suppression of the N2 *tmrl-1* toxin

**Figure 4.**
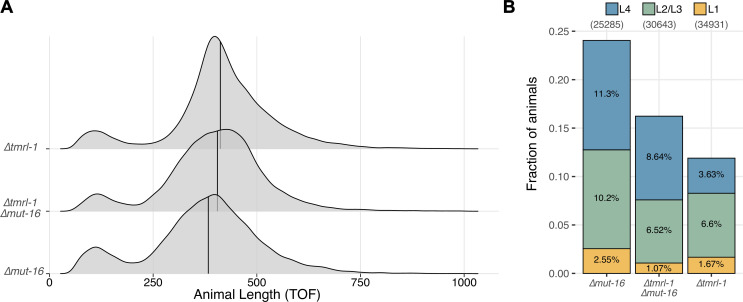
The N2 *tmrl-1* allele contributes to larval arrest in the absence of MUT-16. (**A**) Density plots showing the distribution of animal lengths on the x-axis for the *Δtmrl-1*, Δ*mut-16*, and the *Δtmrl-1; ∆mut-16* double knockout lines. The distribution of animal lengths is significantly different for all comparisons (Kruskal-Wallis test; p=1.56e-133 for the *∆mut-16* to double knockout comparison, p=7.51e-67 for the ∆*tmrl-1* to double knockout comparison, and p ≈ 0 for the *∆mut-16 to* ∆*tmrl-1* comparison). (**B**) Animal length data from (**A**) were binned to approximate larval stages as described in the methods. Stacked bar charts of the fraction of animals for each developmental stage for the *Δtmrl-1, ∆mut-16*, and the *∆tmrl-1; ∆mut-16* double knockout lines are shown. The fraction of the population is shown on the y-axis for each developmental stage—yellow: L1, green: L2/L3, and blue: L4. The fraction of adults is omitted for clarity, but corresponds to the fraction that brings the total to 1 for each genotype.

A potential mechanism that N2-like strains could employ to suppress the activity of *tmrl-1* is RNA interference (RNAi). RNAi pathways are evolutionarily conserved and can act to silence the expression of potentially deleterious genes (Rogers and Phillips, 2020a). In these pathways, argonaute proteins interact with sRNAs to transcriptionally and post-transcriptionally regulate gene expression. In *C. elegans*, primary sRNAs initiate the amplification of secondary small interfering RNAs (siRNAs) in perinuclear granules known as *Mutator* foci (Uebel et al., 2018; Phillips et al., 2012). MUT-16 is a glutamine/asparagine (Q/N)-rich protein that is required for the formation of *Mutator* foci at the nuclear periphery of germline nuclei (Phillips et al., 2012). Previous work has shown that the N2 *tmrl-1* transcript is heavily targeted by secondary 22G siRNAs, the production of which is dependent on MUT-16 and other *Mutator* foci components (Phillips et al., 2012). Animals in which *mut-16* is disrupted with a *mut-16*(*pk170*) mutation show a 137.7-fold decrease in 22G siRNAs that target *tmrl-1* and a corresponding 23.9-fold increase in the expression level of the gene (Reed et al., 2020; Figure 4—figure supplement 1A–B). Furthermore, high levels of larval arrest occur in mutant strains where *Mutator* foci formation is disrupted, including in *mut-16*(*pk170*) strains (Rogers and Phillips, 2020b). Consistent with this report, we observed in a plate-based assay that ~15% of Δ*mut-16* progeny arrested at various larval stages, and 2% of progeny were *rod*, which is suggestive of derepression of *tmrl-1* in N2. We therefore sought to directly test whether *tmrl-1* derepression contributes to larval arrest in the *mut-16(pk170*) strain. To do so, we compared time-of-flight (TOF) measurements—a proxy for animal length, developmental stage, and growth rate (Andersen et al., 2015)—between a strain with a single knockout of *mut-16* and one with a double knockout of *mut-16* and *tmrl-1* (a strain with a single knockout of *tmrl-1* served as a negative control). We observed a reduction in TOF and an increase in the fraction of worms in larval stages in the *mut-16* knockout strain, and these effects were partially rescued in the double knockout strain (Figure 4; Supplementary file 8). These results indicate that the reduced growth rate observed in the *mut-16* knockout strain is partially mediated by the presence of the N2 *tmrl-1* allele, likely because *tmrl-1* is derepressed in *mut-16* knockout strains.

**Figure 4—figure supplement 1.**
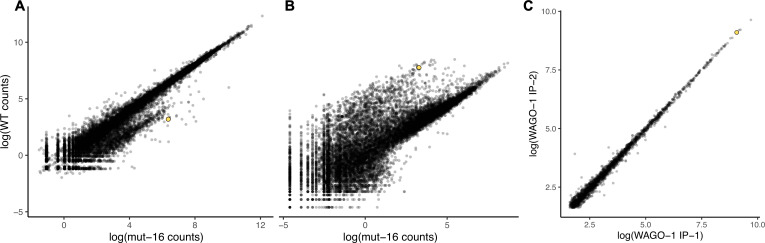
Small RNAs (sRNAs) are involved in the suppression of the N2 tmrl-1 allele. The (**A**) mRNA and (**B**) sRNA abundance is shown for Δ*mut-16* on the x-axis and WT on the y-axis (Reed et al., 2020). The N2 *tmrl-1* (*B0250.8*) allele is represented as a yellow dot. (**C**) sRNA abundance of WAGO-1-associated sRNAs for two independent replicates (Seroussi et al., 2023).

**Figure 4—figure supplement 2.**
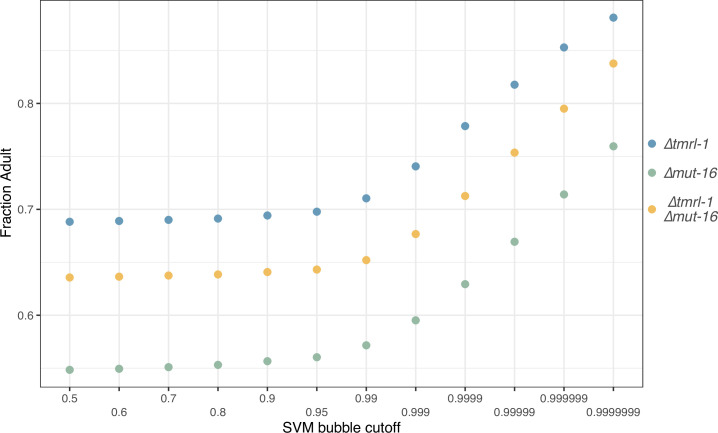
SVM bubble detection for COPAS biosort. The fraction of individual worms that developed to adults is shown on the y-axis for increasingly strict SVM cutoffs that differentiate worms from bubbles on the x-axis. The most strict cutoff of 0.9999999 was used to generate Figure 4.

Amplification of 22G siRNAs can be initiated by different primary sRNAs, including ERGO-1- and ALG-3/4-dependent 26G siRNAs and PRG-1/2-dependent 21U piRNAs. Given that production of MUT-16-dependent 22G siRNAs can be initiated by multiple independent pathways, we queried published sequencing data for sRNAs that are complementary to the N2 *tmrl-1* allele (Makeyeva et al., 2021). This search identified multiple sRNAs that bind throughout the length of the N2 *tmrl-1* transcript. All but one of these sRNAs were not dependent on the argonautes in the queried datasets. We identified one PRG-1-dependent sRNA with a binding site just downstream of two predicted piRNAs, 21ur-8336 and 21ur-14170, which suggests that piRNA recognition of the N2 *tmrl-1* transcript might be involved in its regulation (Wu et al., 2018; Zhang et al., 2018). In support of this hypothesis, sRNA sequencing of PRG-1-bound piRNAs identified several piRNAs that target the N2 *tmrl-1* transcript (21ur-8336, 21ur-2794, 21ur-2025, 21ur-9583, 21ur-5840, 21ur-4143) (Tang et al., 2016; Seroussi et al., 2023). In line with these observations, 22G siRNAs that target the N2 *tmrl-1* transcript are significantly downregulated in *prg-1*(*n4357*) gonads as compared to wild type (fold change –17.1; adjusted p-value < 2.2e-16) (Reed et al., 2020). The depletion of these PRG-1-dependent siRNAs coincides with a 10.3-fold increase in expression of the N2 *tmrl-1* transcript in *prg-1*(*n4357*) gonads (Reed et al., 2020). PRG-1-dependent 22G siRNAs produced in the *Mutator* foci interact with the WAGO-1 argonaute in P-granules to silence transcripts (Gu et al., 2009). Recent work has shown that the *N2 tmrl-1* transcript-derived sRNAs co-immunoprecipitated with WAGO-1, providing additional evidence that this transcript is regulated by the endogenous RNAi machinery (Seroussi et al., 2023; Figure 4—figure supplement 1C). Taken together, these observations suggest that strains with the N2-like haplotype suppress *tmrl-1* toxicity through post-transcriptional silencing mediated by MUT-16-dependent 22G siRNAs that are partially dependent on PRG-1 activity.

## Discussion

We identified a novel TA element in *C. elegans* that consists of two genes, *tmrl-1* and *amrl-1*, which encode a maternally deposited toxin and a zygotically expressed antidote, respectively. Unlike the previously characterized *C. elegans* toxins, PEEL-1 and SUP-35, which induce embryonic lethality in susceptible strains, TMRL-1 induces *rod*-like larval lethality. The delayed onset of lethality suggests that the *tmrl-1* transcript is sequestered from translation and degradation throughout embryogenesis and into the early larval stages. This hypothesis is supported by our observations that *tmrl-1* mRNA is distributed across all cells in early embryonic development but is present only in the Z2/Z3 germ cells in older embryos and L1 larvae. While *tmrl-1* has no detectable homology across all sequence databases and only a very low-confidence protein structure prediction, the induction of the *rod* phenotype by TMRL-1 in susceptible strains suggests that it disrupts osmoregulation in the absence of AMRL-1. The *rod* phenotype is caused by fluid filling of the *C. elegans* pseudocoelom and has been observed after laser and genetic ablation of the excretory canal cell, duct cell, pore cell, or CAN neurons (Nelson and Riddle, 1984; Forrester and Garriga, 1997; Liégeois et al., 2007), which suggests that these cells are affected by TMRL-1.

A unique feature of the *tmrl-1/amrl-1* element is that three distinct haplotypes of this locus exist across the *C. elegans* population. The XZ1516-like haplotype that we originally identified in two crosses is a canonical TA element comprising two linked genes that encode toxin and antidote proteins. The NIC195-like haplotype represents a snapshot of an expected evolutionary trajectory for a TA element, in which the toxin is lost through mutation and the antidote is no longer needed to counteract the toxin. This view is supported by the absence of a toxin-like gene in these strains and the accumulation of mutations in the NIC195 version of the antidote that have reduced its ability to counteract the TMRL-1 toxin. These two haplotypes are present in 7% of the known *C. elegans* strains, while the remaining 93% of strains have the N2-like haplotype.

The N2 version of *tmrl-1* is the most divergent one-to-one ortholog between the N2 and XZ1516 genomes. It is important to note that the two orthologs are hyper-divergent at both the nucleotide and the amino acid levels, as indicated by extremely high dN (0.56) and dS (1.77) values and a dN/dS ratio of 0.32. This value of dN/dS is indicative of purifying selection on the protein sequence, in line with our results, which show that the N2 version of *tmrl-1* has retained its toxicity. The elevated dN and dS values give implausibly long estimates for the divergence time between these two alleles and suggest that positive selection has been driving the diversification of this gene at the nucleotide level. The absence of an intact version of the antidote gene on this haplotype raised the question of how strains which carry it neutralize the toxin and prompted us to look for an alternative mechanism.

A key difference between the N2 and XZ1516 *tmrl-1* transcripts is the presence of several piRNA binding sites across the N2 transcript. These piRNA binding sites likely enable PRG-1 binding to the N2 *tmrl-1* transcript in P granules before the transcript is shuttled to the *Mutator* foci, where 22G siRNAs are produced by *Mutator* class genes (Bagijn et al., 2012; Ashe et al., 2012; Lee et al., 2012; Shirayama et al., 2012; Sundby et al., 2021). The 22G siRNAs that target the *N2 tmrl-1* transcript are among the most abundant transcript-specific 22G siRNAs in the N2 genome, and the production of these 22G siRNAs is dependent on both PRG-1 and MUT-16 (Phillips et al., 2012; Reed et al., 2020). We show that developmental delay phenotypes associated with MUT-16 mutants are partially rescued by the removal of the N2 *tmrl-1* gene, suggesting that this gene is likely functional but highly suppressed in wild-type animals by a mechanism that depends on MUT-16. Taken together, our results suggest that most *C. elegans* strains encode a toxic *tmrl-1* gene that is constitutively silenced by unlinked sRNA machinery. While it is impossible to reconstruct the series of events that led to suppression of a toxin by this mechanism, it is likely that small-RNA-mediated suppression of *tmrl-1* arose prior to the loss of the antidote *amrl-1* in the N2 clade. This scenario is reminiscent of the *Stellate* and *Dox* meiotic drive systems in *Drosophila*, in which small-RNA-encoding genes that are unlinked to their target genes are required to downregulate their respective targets to prevent sex ratio distortions in progeny (Vedanayagam, 2025) and can act as reproductive barriers (Phadnis and Orr, 2009; Bladen et al., 2024). It remains unclear why the N2 *tmrl-1* has not been lost, but we speculate that the divergent *tmrl-1* allele has been maintained in N2-like strains because of a yet-to-be-discovered role it plays in *C. elegans* biology.

## Materials and methods

### Strain maintenance

Unless otherwise specified, all strains were propagated at 20°C on a modified nematode growth medium (NGMA) containing 1% agar and 0.7% agarose and fed *Escherichia coli* strain OP50 (Andersen et al., 2014). Strain names and genotypes can be found in Supplementary file 1.

### Plasmids

Plasmid descriptions can be found in Supplementary file 2. All plasmids generated in this study (with the exception of pWM17) were assembled using gene sequences ordered from IDT (gBlocks) and plasmid backbones (ordered from Addgene or derived from an existing Addgene plasmid) using the NEB Gibson Assembly Master Mix (#E2611). Gibson assemblies were transformed into the assembly mix into DH5α competent cells (NEB #C2987H). Plasmid backbones were digested with restriction enzymes and purified using QIAGEN QIAquick Gel Extraction Kit (#28706). The gBlocks ordered from IDT, the restriction enzymes used, and the primers used to add homology arms to backbones can be found in Supplementary file 3. pWM17 was generated using the NEB Q5 site-directed mutagenesis kit (#E0554S) using pWM11 as the template and primers oZ288 and oZ289. All plasmids were purified using the Invitrogen PureLink HQ Mini Plasmid DNA Purification Kit (#K210001) and verified using whole plasmid sequencing with primordium.

### Multi-generational cross

QX2538 (*fog-2*(qq212[P17stop]) in XZ1516), QX2539 (*fog-2*(qq212[P17stop]) in QX1211), and QX2327 (qqIr39[fog-2(q71), N2>DL238] V) were used to generate the cross populations. QX2538 males were used to start the QX2538 × QX2539 cross population and QX2327 males were used to start the QX2327 × QX2538 cross population. Crosses were amplified on 10 cm NGMA plates until approximately 20,000 worms were obtained, at which point the population size was maintained. For each generation, worms were washed off the plates using M9, bleach synchronized, and arrested overnight as L1s. The following day, 20,000 L1s were plated across ten 10 cm plates at ~2000 worms/plate. The crosses were propagated until the 10th generation of intercrossing. Multiple timepoints were saved for sequencing by freezing a pellet of several thousand worms. For generation 10, DNA libraries were generated using the Illumina Nextera XT DNA Library Preparation Kit (#FC-131-1024) and whole genome sequenced using Illumina NextSeq2000 P1 reagents (20074933). For generation 4, allele frequencies were inferred as previously described (Ben-David et al., 2021a).

### Crosses

Descriptions of the crosses and their phenotypes can be found in Supplementary file 4. Workflow for crosses varied slightly depending on the use of fluorescent males. For all crosses, males and L4 hermaphrodites were allowed to mate 24–36 hr before potentially mated hermaphrodites were singled out onto fresh plates. For crosses set up with wild-type males, only progeny from plates with a 50:50 male to hermaphrodite ratio were used for subsequent crosses. For crosses set up with fluorescent males, only progeny with the fluorescent marker were used for subsequent crosses. For self crosses, ~10 hermaphrodite cross progeny were transferred to fresh agar plates and allowed to lay embryos for ~24 hr. For parental backcrosses, ~5 L4 cross progeny were allowed to mate with the designated parent for 24–36 hr before potentially mated hermaphrodites were singled out onto fresh plates and laid embryos for ~24 hr. For all crosses, a defined number of embryos were transferred to fresh plates and allowed to hatch overnight. The following day, the number of arrested larvae were counted.

### NIL construction

The QX2500 and QX2501 NILs were constructed as previously described (Zdraljevic et al., 2023). Briefly, QX2500 was constructed by genotyping F2 progeny of a QX1211 × XZ1516 cross. DNA from F2/F3 progeny was amplified using primer pairs: oZ50-51 and oZ52-53, which enabled us to identify recombinants within a defined genomic region. Recombinant progeny identified with these primer pairs were recovered and backcrossed to QX1211 for six generations. We generated QX2501 using Cas9-induced non-homologous recombination to induce strand exchange (Zdraljevic et al., 2023). Young adult QX2500/QX1211 heterozygotes were injected with four target guide RNAs (gRNAs) (gSZ63, gSZ65, gSZ68, and gSZ70), the *dpy-10* gRNA, and repair template (see Cas9 injections). We transferred F2 *rol* animals to 96-well plates, allowed them to self, and identified recombinant individuals using oZ66-67 and oZ64-65. We identified and isolated a recombinant that we named QX2501, which contains the QX1211 genotype from V:1–21,536,657, followed by a deletion spanning V:21,536,657–21,547,827, and the XZ1516 genotype from V:21,547,828–22,058,188. All genotyping primers can be found in Supplementary file 5.

### Fine-mapping the element

We previously described the construction of the 10 gene CINR-generated NIL (Zdraljevic et al., 2023). Briefly, young adult QX1211/QX2501 were injected with gSZ71, and progeny were genotyped with oZ80-82-86 and oZ64-65 to identify individuals that underwent a loss of heterozygosity event.

### Injection workflow

The following workflow was used for all injections (candidate gene KOs, antidote rescue, and tet-inducible system). The day before injections, L4 animals were transferred to fresh 6 cm NGMA plates and allowed to develop overnight. The following day, young adults were injected and transferred to a fresh 6 cm NGMA plate to recover. The injected animals were singled to fresh 6 cm NGMA plates approximately 16 hr after injections and monitored for the co-injection phenotype.

### Candidate gene knockouts with CRISPR/Cas9

All gRNAs were designed to target the XZ1516 genome using the *multicrispr* R package (Zdraljevic et al., 2023; Bhagwat et al., 2020; R Development Core Team, 2017). gRNAs were purchased from Synthego as synthetic spacer-scaffold fusions. gRNAs were resuspended in 30 µl of water (50 µM) and stored at –20°C. Cas9 was purchased from IDT (cat #1081059) and stored in single-use aliquots (0.5 µl to 5 µg Cas9) at –80°C. Injection mixtures were made on ice immediately before use. For each mixture, gRNAs (including oZ30; *dpy-10*) were added to a Cas9 aliquot and incubated at 37°C for 10 min. Then the *dpy-10* single-stranded oligodeoxynucleotide (ssODN) repair template (oZ31) and water were added to a final volume of 20 µl. The mixture was spun down in a table-top centrifuge at maximum speed for 5 min, and 10 µl was taken off of the top to use in injections. The final concentrations in the injection mixtures were: 1.5 µM of Cas9, 4.45 µM gRNA (with each gRNA represented equally), and 0.5 µM of the ssODN repair template. gRNA and repair template sequences can be found in Supplementary file 6.

### Antidote rescue

The antidote rescue injection mixture was made on the same day as injections. The mixture was made at room temperature, spun down at maximum speed for 5 min, and 10 µl was taken off of the top to use in injections. The final concentrations were: 30 ng/µl pWM4, ng/µl pCFJ104, and 65 ng/µl GeneRuler 1 kb DNA ladder (cat #SM0311). Both DL238 and XZ1516 were injected. Progeny of injected animals that expressed the co-injection marker pCFJ104 were isolated and propagated. DL238 co-inj (+) animals were crossed to XZ1516 WT, and XZ1516 co-inj (+) animals were crossed to DL238 WT to assess lethality. In addition to the previously described cross workflow, we also took note of whether or not dead animals inherited the array (as indicated by co-inj (+)).

### Long-read direct RNA sequencing

We extracted and purified RNA (MasterPure #MC85200) from a mixed stage culture of XZ1516. A TapeStation confirmed the high quality of the sample (RINe = 9.3). We prepared long-read RNA libraries using Oxford Nanopore library prep kit #SQK-RNA002 on the purified XZ1516 RNA. Libraries were run on a MinION Flow Cell (R9.4.1), bases were called using *guppy*, and the long reads were aligned to the XZ1516 genome using *minimap2* (Li, 2018).

### Tet-inducible system

We used a tet-inducible system to test the toxicity of several constructs (Mao et al., 2019). The system consists of three plasmids: a tet promoter driving the CDS of interest, a tet activator (TC374), and a tet GFP (TC358). Injection mixtures were made at room temperature, spun down at maximum speed for 5 min, and 10 µl was taken off of the top to use in injections. The final concentrations were: 5 µM expression construct (pWM8, 11, 12, 17, or 21), 5 ng/µl TC374, 5 ng/µl TC358, 5 ng/µl pCFJ104, and 80 ng/µl GeneRuler 1 kb DNA ladder (cat #SM0311). DL238 was injected with pWM11, pWM12, and pWM17 to test *tmrl-1* toxicity. DL238, XZ1516, and N2 were injected with pWM21 to test *B0250.8* toxicity and with pWM8 as a negative control for all tet-inducible injections. Progeny of injected animals that expressed the co-injection marker pCFJ104 were isolated and propagated. We assessed toxicity of the expression construct of interest through induction on NGM plates with 0.17% doxycycline hyclate (Sigma # D9891), seeded with HT115 bacteria. Most often pCFJ104 (+) gravid worms were bleached onto dox and control plates and phenotyped 48 hr later.

### Microscopy

RNA FISH: The sequence corresponding to the *tmrl-1* transcript was uploaded to Stellaris’s probe design feature, which yielded a mixture of 25 RNA probes, each 19 nucleotides long (Biosearch Technologies). Probes were labeled with CAL Fluor Red 610. XZ1516, DL238, and QX2513 were grown in liquid cultures to amplify the populations. Gravid adults were bleached from liquid cultures, and embryos were fixed immediately or allowed to arrest in liquid overnight. Embryos and arrested L1s were prepared according to the Stellaris RNA FISH Protocol for *C. elegans* with the following modifications. Rather than using chambered coverglass, the hybridization and subsequent wash steps were conducted on fixed embryos or larvae in a microcentrifuge tube. ProLong Glass Antifade Mountant (Thermo Fisher #P36982), rather than Vectashield mounting medium, was added to prepared samples before mounting on slides. Images were taken using a NIKON Eclipse Ti2 widefield microscope with a 100×/1.45 plan apochromat lambda D oil objective, a Photometrics Prime 95B large field of view monochrome fluorescence camera, and a SpectraIII/Celesta/Ziva, MultiLaser (SpectraIII/Lida) light source. The following filters were used: UV excitation 365 nm, emission 435 nm, and mCherry excitation 514 nm, emission 543 nm. z-Stack images were taken with a 0.2 µm step size. Image analysis was performed in NIS Elements AR5.4.

### Population analysis of the TA element

To determine the strain relatedness of the *C. elegans* population, we extracted variants in the region surrounding the B0250.4 and B0250.8 (V:20454811–20473950) from the CeNDR VCF (version 20220216). After subsetting the VCF, we used the *vcf2dist* and *dist2tree* functions in the *fastreeR* R package to generate the relatedness dendrogram.

We used the R package *orthologr* to calculate nucleotide and amino acid identity and dS (Drost et al., 2015). We calculated dS across all orthologs between the N2 and XZ1516 genomes using all of the methods available in *orthologr*. We used the following formula to calculate divergence time: T = (dS − πanc)/(2μ) (Gillespie and Langley, 1979), as previously described (Thomas et al., 2015).

### Mut-16 phenotyping

QX2537, QX2532, and QX2534 were propagated on 10 cm NGMA plates until gravid. The three strains were bleached, and L1s were arrested overnight shaking at 20°C and 180 rpm in K media (Zdraljevic et al., 2017; Boyd et al., 2012). The next day, samples were fed HB101 *E. coli* at a final concentration of OD10 and allowed to grow for 48 hr, shaking at 20°C and 180 rpm. Prior to scoring strains on the COPAS BIOSORT using the sample cup, sodium azide was added at a final concentration of 50 mM (Andersen et al., 2015). The resulting data was loaded into R for analysis (R Development Core Team, 2017). Objects with a TOF value greater than 60 and less than 1000 were retained for analysis. We used a pre-trained SVM to detect bubbles in the dataset (*COPASutils::bubbleSVMmodel_noProfiler*; threshold = 0.9999999) (Shimko and Andersen, 2014). Larval stages were defined by TOF (60<TOF<90=L1; 90<TOF<200=L2/L3; 200<TOF<300=L4; 300<TOF<1000=adult). We note that the conclusions drawn from this experiment did not depend on the SVM threshold used (Figure 4—figure supplement 2).

## Data Availability

All data are available in the manuscript or the supplementary materials. All materials are available by request. Additional datasets have been added to Dryad: https://doi.org/10.5061/dryad.3ffbg79tq. The following dataset was generated: ZdraljevicS Walter-McNeillL BruniG BloomJ LeightonD MarquezH AlexanderN 2024Divergent *C. elegans* toxin alleles are suppressed by distinct mechanismsDryad Digital Repository10.5061/dryad.3ffbg79tqPMC1346114742578454

## Additional files

Supplementary file 1. Strains.

Supplementary file 2. Plasmids.

Supplementary file 3. Plasmid construction.

Supplementary file 4. Crosses.

Supplementary file 5. Oligos.

Supplementary file 6. Guide RNAs (gRNAs) and repair.

Supplementary file 7. Inducible construct phenotypes.

Supplementary file 8. COPAS biosort data.

MDAR checklist
