# Supplementary material for: Divergent C. elegans toxin alleles are suppressed by distinct mechanisms
Source: eLife. 2026 Aug 11;14:RP106269. doi: 10.7554/eLife.106269 (PMC13461147; doi:10.7554/eLife.106269)
Supplement: Peer review history

# Editor report: eLife Assessment

**DOI:** 10.7554/eLife.106269.3.sa0

**Authors:** Vincent J Lynch

**Affiliations:** University at Buffalo, State University of New York United States

**Keywords:** Important, Solid

This **important** study identifies a new toxin/antidote (T/A) system in the model nematode *C. elegans*. These results suggest there are alternative mechanisms to neutralize selfish genetic elements. The authors present **solid** data that robustly support their central conclusion. This work will be of broad interest to investigators in evolutionary biology and reproductive biology.

# Referee report: Reviewer #1 (Public review)

**DOI:** 10.7554/eLife.106269.3.sa1

Summary:

The article by Zdraljevic et al. reports the discovery of a third toxin-antidote (TA) element in *C. elegans*, composed of the genes mll-1 (toxin) and smll-1 (antidote). Unlike previously characterized TA systems in *C. elegans,* this element induces larval arrest rather than embryonic lethality. The study identifies three distinct haplotypes at the TA locus, including a hyper-divergent version in the standard laboratory strain N2, which retains a functional toxin but lacks a functional antidote. The authors propose that small RNA-mediated silencing mechanisms, dependent on MUT-16 and PRG-1, suppress the toxicity of the divergent toxin allele. This work provides insights into the evolutionary dynamics of TA elements and their regulation through RNA interference (RNAi).

Overall, there are many things to like about this paper and only a few small quibbles, which will not require more than a little rewriting or relatively minor analyses.

Strengths of the Paper:

(1) The discovery of a maternally deposited TA element with delayed toxicity due to delayed mRNA translation of the maternally deposited toxin mRNA is a significant addition to the literature on selfish genetic elements in metazoans.

(2) Identifying three haplotypes at the TA locus provides a snapshot of potential evolutionary trajectories for these elements, which are often inferred but rarely demonstrated in naturally occurring strains. The genomic analysis of 550 wild isolates contextualizes the findings within natural populations, revealing geographic clustering and evolutionary pressures acting on the TA locus.

(3) The study employs various techniques, including CRISPR/Cas9 knockouts, FISH, long-read RNA sequencing, and population genomics. The use of inducible systems to confirm toxicity and antidote functionality is particularly robust. This multifaceted approach strengthens the validity of the findings.

(4) The authors provide compelling evidence that small RNA pathways suppress toxin activity in strains lacking a functional antidote. This highlights an alternative mechanism for neutralizing selfish genetic elements.

Comments on revised version.

The authors have addressed all my (relatively minor) comments from the first round of reviews. However, the most substantial comments came from Reviewer 2, mostly focused on the conclusions that "Multiple lines of evidence suggest that the N2 tmrl-1 allele is recognized by piRNAs, leading to MUT-16-dependent 22G siRNA production and post-transcriptional silencing of the transcript." This is beyond my expertise to fully evaluate what is state-of-the-art in terms of acceptable evidence, so I will defer to Reviewer #2 for this.

# Referee report: Reviewer #2 (Public review)

**DOI:** 10.7554/eLife.106269.3.sa2

Summary:

In the manuscript by Walter-McNeill, Kruglyak and team, the authors provide solid evidence of another toxin-antidote (TA) system in *C. elegans*. Generally, TA systems involve selfish and linked genetic elements, one encoding a toxin that kills progeny inheriting it, unless an antidote (the second element) is also present. Currently, only two TA systems have been characterized in this species, pointing to the importance of identifying new instances of such systems to understand their transmission dynamics, prevalence, and functions in shaping worm populations.

The manuscript has been improved in some aspects upon revision. We remain enthusiastic for the overall findings and the identification of a new toxin/anti-toxin system and note that the strengths and weaknesses we detailed previously remain. We reiterate our critique regarding the strength of conclusions that can be made about small RNA pathway regulation based on meta-analysis of other datasets. While we agree that the observations presented are suggestive of small RNA regulation, likely due to piRNA targeting and subsequent 22G-RNA regulation, until these hypotheses are tested experimentally in the future by mutation of the piRNA target sites, testing ago/piRNA pathway and other 22G-RNA pathway mutants for tmrl-1 expression, etc., we think it is important to use precise language in presenting the conclusions. In particular, the abstract states:

"Multiple lines of evidence suggest that the N2 tmrl-1 allele is recognized by piRNAs, leading to MUT-16-dependent 22G siRNA production and post-transcriptional silencing of the transcript. The N2 haplotype represents the first naturally occurring unlinked toxin-antidote system where the toxin is post-transcriptionally suppressed by endogenous small RNA pathways."

We therefore recommend moderating this statement to "...is likely to be post-transcriptionally suppressed by endogenous small RNA pathways."

Previously noted strengths and weaknesses remain relevant to this revision.

Strengths:

This novel TA system (mll-1/smll-1) was identified on LGV in wild *C. elegans* isolates from the Hawaiian Islands, by crossing divergent strains and observing allele frequency distortions by high throughput genome sequencing after 10 generations. These allele frequency distortions were subsequently confirmed in another set of crosses with a separate divergent strain, and crosses of heterozygous males or hermaphrodites resulted in a pattern of L1 lethality in progeny (with a rod arrest phenotype) that suggested the maternal transmission of this TA system from the XZ1516 genetic background. By elegantly combining the use of near-isogenic lines, CRISPR editing to generate knock-outs, and a transgene rescue of the antidote gene, the authors identified the genes encoding the toxin and the antidote, which they refer to as mll-1 and smll-1. Moreover, the specific mll-1 isoform responsible for the production of the toxin was identified and mll-1 transcripts were observed by FISH in early and late embryos, as well as in larvae. Inducible expression of the toxin in various strains resulted in larval arrest and rod phenotypes. The authors then characterized the genetic variation of 550 wild isolates at the toxin/antidote region on LGV and distinguished three clades: (1) one with the conserved TA system, (2) one having lost the toxin and retaining a mostly functional antidote, and (3) one having lost the antidote and retaining a divergent yet coding toxin (this includes the reference strain Bristol N2, in which the homologous toxin gene has acquired mutations and is known as B0250.8). Further, the authors show that this region is under positive selection. These data are compelling and provide very strong evidence of a new TA system in this species.

Weaknesses:

The question remained as to how one clade, including N2, could retain the toxin gene but not possess a functional antidote. In the second part of the manuscript, the authors hypothesized that small RNA targeting (RNAi) of the toxin transcript could provide the necessary repression to allow worms to survive without the antidote. Through a meta-analysis of multiple small RNA datasets from the literature, the authors found evidence to support this idea, in which the toxin transcript is targeted by 22G siRNAs whose biogenesis is dependent on the Mutator foci protein, MUT-16. They note that from previous studies, mut-16 null mutants displayed a varied penetrance of larval arrest. In their own hands, mut-16 mutants displayed 15% varied larval arrest and 2% rod phenotypes. In an attempt to link B0250.8 to mut-16/siRNAs, they made a double mutant and examined body length as a proxy for developmental stage. Here, they observed a partial rescue of the mut-16 size defect by B0250.8 mutation. Finally, the authors also highlight data from further meta-analysis which predicts the recognition of B0250.8 by several piRNAs. Also based on existing data from the literature, the authors link loss of Piwi (PRG-1), which binds piRNAs, to a depletion of 22G-RNAs targeting B0250.8 and an upregulation of B0250.8 expression in gonads, suggesting that piRNAs are the primary small RNAs that target B0250.8 for down-regulation. The data in this portion of the manuscript are intriguing, but somewhat incomplete, as they are based on little primary experimentation and a collection of different datasets (which have been acquired by slightly different methods in most cases). This portion of the study would require subsequent experimentation to firmly establish this mechanistic link. For example, to be able to claim that "the N2 toxin allele has acquired mutations that enable piRNA binding to initiate MUT-16-dependent 22G small RNA amplification that targets the transcript for degradation" the identified piRNA sites should be mutated and protein and transcript levels analysed in wild-type and in the strain with mutated piRNA sites. At a minimum, the protein levels in wild-type and mut-16, prg-1, and/or wago-1 mutants should be measured by western blot and/or by live imaging (introducing a GFP or some other tag to the endogenous protein via CRISPR editing) to show that the toxin is not accumulated as a protein in wt, but increases in levels in these mutants. mRNA levels in Fig S5A suggest there is still some expression of the B0250.8 transcript in a wild type situation.

Comments on revised version.

We have no further recommendations for the authors, other than those provided above.

# Author comment: Author response

**DOI:** 10.7554/eLife.106269.3.sa3

**Authors:** Stefan Zdraljevic, Laura Walter-McNeill, Giancarlo N Bruni, Joshua S Bloom, Daniel HW Leighton, Heriberto Marquez, Noah Alexander, Leonid Kruglyak

**Affiliations:** University of California, Los Angeles Los Angeles United States; MIT University Cambridge United States; Howard Hughes Medical Institute Chevy Chase United States; Howard Hughes Medical Institute CHevy Chase United States; Howard Hughes Medical Institute Chevy Chase United States; Howard Hughes Medical Institute Chevy Chase United States; University of California, Los Angeles Los Angeles United States; Howard Hughes Medical Institute Maryland United States

The following is the authors’ response to the original reviews.

We incorporated Reviewer #2’s suggestion to change the name of mll-1 because of overlap with a human gene. We used the updated gene names in our responses below to minimize confusion. Below are the updated gene names for the toxin-antidote system we described.

*tmrl-1* - Toxin-induced Maternal Rod Lethality (formerly mll-1). After we establish that B0250.8 is also a toxin, we refer to this gene as the “N2 *tmrl-1* allele”.

*amrl-1* - Antidote of Maternal Rod Lethality (formerly smll-1)

**Public Reviews:**

**Reviewer #1 (Public review):**

Summary:

The article by Zdraljevic et al. reports the discovery of a third toxin-antidote (TA) element in *C. elegans*, composed of the genes mll-1 (toxin) and smll-1 (antidote). Unlike previously characterized TA systems in *C. elegans*, this element induces larval arrest rather than embryonic lethality. The study identifies three distinct haplotypes at the TA locus, including a hyper-divergent version in the standard laboratory strain N2, which retains a functional toxin but lacks a functional antidote. The authors propose that small RNA-mediated silencing mechanisms, dependent on MUT-16 and PRG-1, suppress the toxicity of the divergent toxin allele. This work provides insights into the evolutionary dynamics of TA elements and their regulation through RNA interference (RNAi).

Overall, there are many things to like about this paper and only a few small quibbles, which will not require more than a little rewriting or relatively minor analyses.

Strengths:

(1) The discovery of a maternally deposited TA element with delayed toxicity due to delayed mRNA translation of the maternally deposited toxin mRNA is a significant addition to the literature on selfish genetic elements in metazoans.

(2) Identifying three haplotypes at the TA locus provides a snapshot of potential evolutionary trajectories for these elements, which are often inferred but rarely demonstrated in naturally occurring strains. The genomic analysis of 550 wild isolates contextualizes the findings within natural populations, revealing geographic clustering and evolutionary pressures acting on the TA locus.

(3) The study employs various techniques, including CRISPR/Cas9 knockouts, FISH, long-read RNA sequencing, and population genomics. The use of inducible systems to confirm toxicity and antidote functionality is particularly robust. This multifaceted approach strengthens the validity of the findings.

(4) The authors provide compelling evidence that small RNA pathways suppress toxin activity in strains lacking a functional antidote. This highlights an alternative mechanism for neutralizing selfish genetic elements.

Weaknesses:

(1) The introduction focuses strongly (for good reason) on bacterial TA systems and then jumps to TA systems in *C. elegans*. It's unclear why TA systems in other eukaryotes are not discussed.

We briefly introduced bacterial TA systems because of their ubiquitousness and focused on *C. elegans* TA systems. We chose certain aspects of previously described Caenorhabditis TA elements that were relevant to the narrative we presented. Furthermore, we have extensively reviewed TA systems previously and have added a citation to that review in the revised manuscript (Burga et al. 2020).

(2) Similarly, there is a missed opportunity to discuss an analogy between the suppressor mechanism discovered here and the hairpin RNA suppressors of meiotic drive identified by Eric Lai and colleagues. Discussing these will provide a fuller context of the present study's findings and will not affect their novelty.

Thank you for pointing this out. We added a mention of the Stellate and Dox systems in our discussion.

(3) While the evidence for RNAi-mediated suppression is strong, the claim that positive selection drove diversification at piRNA binding sites requires further discussion and clarification. The elevated dN and dS are unusual (how unusual relative to other genes in vicinity? What is hyper-divergent statistically speaking?), but there is no a priori reason that there would be selection on piRNA binding sites within the mll-1 transcript to facilitate its recognition by endogenous RNAi machinery; what is the selective pressure for mll-1 to do so? Most TA systems would like to avoid being suppressed by the host. One cannot make the argument that this was motivated by the loss of the antidote because the loss of the antidote would be instantly suicidal, so the cadence of events described requiring hypermutation of the mll-1 transcript does not work.

We largely agree with the reviewer’s point, which we believe is based on the following sentence in the discussion: “We propose that positive selection for piRNA binding sites in the tmrl-1 transcript drove the diversification of this gene toward the N2 version.” We have removed this argument from the discussion in the revised manuscript.

**Reviewer #2 (Public review):**

Summary:

In the manuscript by Walter-McNeill, Kruglyak, and team, the authors provide solid evidence of another toxin-antidote (TA) system in *C. elegans*. Generally, TA systems involve selfish and linked genetic elements, one encoding a toxin that kills progeny inheriting it, unless an antidote (the second element) is also present. Currently, only two TA systems have been characterized in this species, pointing to the importance of identifying new instances of such systems to understand their transmission dynamics, prevalence, and functions in shaping worm populations.

Strengths:

This novel TA system (mll-1/smll-1) was identified on LGV in wild *C. elegans* isolates from the Hawaiian islands, by crossing divergent strains and observing allele frequency distortions by high-throughput genome sequencing after 10 generations. These allele frequency distortions were subsequently confirmed in another set of crosses with a separate divergent strain, and crosses of heterozygous males or hermaphrodites resulted in a pattern of L1 lethality in progeny (with a rod arrest phenotype) that suggested the maternal transmission of this TA system from the XZ1516 genetic background. By elegantly combining the use of near-isogenic lines, CRISPR editing to generate knock-outs, and a transgene rescue of the antidote gene, the authors identified the genes encoding the toxin and the antidote, which they refer to as mll-1 and smll-1. Moreover, the specific mll-1 isoform responsible for the production of the toxin was identified and mll-1 transcripts were observed by FISH in early and late embryos, as well as in larvae. Inducible expression of the toxin in various strains resulted in larval arrest and rod phenotypes. The authors then characterized the genetic variation of 550 wild isolates at the toxin/antidote region on LGV and distinguished three clades: (1) one with the conserved TA system, (2) one having lost the toxin and retaining a mostly functional antidote, and (3) one having lost the antidote and retaining a divergent yet coding toxin (this includes the reference strain Bristol N2, in which the homologous toxin gene has acquired mutations and is known as B0250.8). Further, the authors show that this region is under positive selection. These data are compelling and provide very strong evidence of a new TA system in this species.

Weaknesses:

The question remained as to how one clade, including N2, could retain the toxin gene but not possess a functional antidote. In the second part of the manuscript, the authors hypothesized that small RNA targeting (RNAi) of the toxin transcript could provide the necessary repression to allow worms to survive without the antidote. Through a meta-analysis of multiple small RNA datasets from the literature, the authors found evidence to support this idea, in which the toxin transcript is targeted by 22G siRNAs whose biogenesis is dependent on the Mutator foci protein, MUT-16. They note that from previous studies, mut-16 null mutants displayed a varied penetrance of larval arrest. In their own hands, mut-16 mutants displayed 15% varied larval arrest and 2% rod phenotypes. In an attempt to link B0250.8 to mut-16/siRNAs, they made a double mutant and examined body length as a proxy for developmental stage. Here, they observed a partial rescue of the mut-16 size defect by B0250.8 mutation. Finally, the authors also highlight data from further meta-analysis, which predicts the recognition of B0250.8 by several piRNAs. Also based on existing data from the literature, the authors link loss of Piwi (PRG-1), which binds piRNAs, to a depletion of 22G-RNAs targeting B0250.8 and an upregulation of B0250.8 expression in gonads, suggesting that piRNAs are the primary small RNAs that target B0250.8 for downregulation. The data in this portion of the manuscript are intriguing, but somewhat preliminary and incomplete, as they are based on little primary experimentation and a collection of different datasets (which have been acquired by slightly different methods in most cases). This portion of the study would require subsequent experimentation to firmly establish this mechanistic link. For example, to be able to claim that "the N2 toxin allele has acquired mutations that enable piRNA binding to initiate MUT-16-dependent 22G small RNA amplification that targets the transcript for degradation" the identified piRNA sites should be mutated and protein and transcript levels analysed in wild-type and in the strain with mutated piRNA sites. At a minimum, the protein levels in wild-type and mut-16, prg-1, and/or wago-1 mutants should be measured by western blot and/or by live imaging (introducing a GFP or some other tag to the endogenous protein via CRISPR editing) to show that the toxin is not accumulated as a protein in wt, but increases in levels in these mutants. mRNA levels in Figure S5A suggest there is still some expression of the B0250.8 transcript in a wild-type situation.

We thank the reviewer for their thoughtful assessment of our manuscript, and we appreciate that they recognized that the data linking the small RNA machinery to B0250.8 suppression is intriguing. While the reviewer claims our analysis is preliminary and incomplete, we believe we present an appropriate multi-faceted approach for establishing the small RNA-mediated suppression mechanism we describe.

First, the reviewer states that we rely on “little primary experimentation”. Our primary experiments show that loss of the N2 tmrl-1 allele partially rescues ∆mut-16 developmental delay and arrest phenotypes. Therefore, we provide direct evidence that the N2 tmrl-1 functionally contributes to the ∆mut-16 phenotype. Furthermore, we overexpressed the N2 tmrl-1 allele to show that this gene is a toxin.

It is true that we use previously published datasets to establish a small RNA-mediated mechanism that likely explains our observations. The reviewer suggests that our claims are weakened by relying on a “collection of different datasets (which have been acquired by slightly different methods in most cases)”. We believe instead that evidence collected from multiple labs using an array of different techniques strengthens our conclusions. We show that N2 tmrl-1-targeting small RNAs have been identified across multiple datasets (references 26, 32, 33, 34). Taken together, these datasets support a mechanistic framework for the suppression of the N2 tmrl-1 that involves PRG-1-dependent piRNA binding, MUT-16-dependent 22G siRNA, and the secondary Ago WAGO-1 binding.

The reviewer suggests several experiments, but we do not view them as essential to support our claims.

(a) piRNA site mutatagenesis: we present multiple lines of evidence that the N2 tmrl-1 transcript is post-transcriptionally targeted by small RNAs in a piRNA-mediated manner, not that specific piRNA sites are necessary and sufficient for this silencing. The suggested experiment would be valuable for future work, but is beyond the scope of our study.

(b) Characterization of TMRL-1 protein levels: We agree that this experiment would provide definitive evidence of complete small RNA-mediated suppression of the N2 tmrl-1 transcript. As we explain above, however, we do show that removing the N2 tmrl-1 allele partially rescues the ∆mut-16 growth defect, demonstrating that when this gene’s regulation is disrupted, it induces toxicity. Importantly, we observed no tmrl-1-induced toxicity when we overexpressed a version of this gene with a stop codon, indicating that it acts as a protein.

Finally, the reviewer questions our claim that: "the N2 toxin allele has acquired mutations that enable piRNA binding to initiate MUT-16-dependent 22G small RNA amplification that targets the transcript for degradation."

We agree that this statement is too definitive given our current data. We have revised it to: "Multiple lines of evidence suggest that the N2 tmrl-1 allele is recognized by piRNAs, leading to MUT-16-dependent 22G siRNA production and post-transcriptional silencing of the transcript."

**Recommendations for the authors:**

**Reviewer #1 (Recommendations for the authors):**

(1) The paper suggests that antidote pseudogenization occurred because RNAi replaced its function, but does not explore whether this process is ongoing or complete across all N2-like strains.

We explored this possibility, but we realize that we did not explicitly state so in the manuscript. The B0250.4 (amrl-1) gene is pseudogenized in all strains within the N2 clade. We have modified the following sentence in the results section to explicitly state this observation:

“While the previously described *C. elegans* TA elements are characterized by their absence in susceptible strains (2, 3), all members of the N2-like susceptible clade harbor a divergent allele of tmrl-1 with an intact coding sequence, as well as a pseudogenized version of amrl-1.”

(2) Some figures (e.g., allele frequency distortions) could benefit from additional annotations to guide interpretation. In general, the figures make the reader work harder than they need to.

We attempted to add clarity to figure captions for clarity.

Although mll-1 and smll-1 were identified as toxin and antidote genes, their molecular mechanisms remain unclear and are very interesting.

We agree that identifying the molecular mechanism associated with the toxin and antidote would be of interest, but is beyond the scope of the current paper.

**Reviewer #2 (Recommendations for the authors):**

(1) Because the rod phenotype was important in identifying the TA system, it seems important to include representative images of this phenotype throughout the paper.

We added a supplemental figure showing the resulting self progeny from a QX1211/XZ1516 heterozygote: Fig S1B

(2) In Figure 2A, we were confused as to why there were so few reads of mll-1. We may be misunderstanding something, so could the authors explain this to us? We would have expected more reads of mll-1, given the diagram showing that the breakpoints of the NIL were beyond (closer to the right end of) the mll-1 locus, and the phenotype correlates with the presence of the toxin (frequency of .20 L1 arrest).

The lack of sequencing depth arises because the sequence divergence between QX1211 and XZ1516 is too high to accurately map short sequencing reads derived from QX1211 to the XZ1516 genome. We added the following sentence to the figure caption to add clarity:

“The XZ1516 and QX1211 genome are so diverged that short reads derived from QX1211 don’t align to the XZ1516 genome in the 200 bp windows with no corresponding read depth, as indicated by a lack of a gray bar.”

(3) The use of TOF in Figure 4 as a proxy of animal length instead of directly indicating or measuring animal length hinders the comparison of these results with other studies (i.e., most often in the literature, we see images of worms and measurements of their sizes or use of some other morphological marker to demonstrate the proportion of worms in a particular developmental stage). Nonetheless, we think the approach is clever and certainly enables analysis of a large sample population. However, a wild-type control is missing from these experiments to give a sense of the typical distribution one would expect. Without this, one interpretation of the B0250.8 knock out data shown in B is that loss of B0250.8 results in ~10% arrested larval, which seems higher than would be expected for a wild type N2 strain, and should be explained-but again, if the wild type control showed the same pattern, that would be useful to know. The title for Figure 4 should be revised, as this figure suggests, but does not provide definitive evidence that B0250.8 is suppressed by sRNAs/sRNA pathways. See the next point for providing more definitive data to support this model.

There is a long list of publications that rely on the large particle sorter to infer how growth rate is affected in various mutants and environmental conditions (See Andersen et al. 2015, ref 28 in the manuscript, and the papers that reference this work). As the reviewer pointed out, the use of time of flight, which is simply the amount of time an object obstructs a laser at a constant flow rate, enables accurate measurement of tens of thousands of individual animals for comparison.

The reviewer is correct to point out that without a wild type N2 control, it is impossible to tell what a typical distribution looks like. However, the experiment includes all strains necessary to make the comparisons that enable us to draw the conclusion that the N2 tmrl-1 allele contributes to larval arrest in the absence of MUT-16.

We agree with the reviewers point that this figure does not provide evidence that B0250.8 is suppressed by small RNAs and we have therefore changed the figure title.

The new figure title: The N2 tmrl-1 allele contributes to larval arrest in the absence of MUT-16

(4) To be able to claim that "the N2 toxin allele has acquired mutations that enable piRNA binding to initiate MUT-16-dependent 22G small RNA amplification that targets the transcript for degradation" the identified piRNA sites should be mutated and protein and transcript levels analysed in wild-type and in the strain with mutated piRNA sites. At a minimum, the protein levels in wild-type and mut-16, prg-1, and/or wago-1 mutants should be measured by western blot and/or by live imaging (introducing a GFP or some other tag to the endogenous protein via CRISPR editing) to show that the toxin is not accumulated as a protein in wt, but increases in levels in these mutants. mRNA levels in Figure S5A suggest there is still some expression of the B0250.8 transcript in a wild-type situation.

The reviewer makes several good suggestions for experiments to determine whether the conclusions we make from publicly available high-throughput sequencing datasets apply in our context. However, we disagree that the quoted statement “the N2 toxin allele has acquired mutations that enable piRNA binding to initiate MUT-16-dependent 22G small RNA amplification that targets the transcript for degradation” is not supported by the evidence we present from Reed et al. 2020. The data presented by Reed et al. clearly show that the N2 tmrl-1 transcript is heavily targeted by 22G siRNAs, and that the accumulation of these siRNAs depends on the presence of MUT-16 and PRG-1. The dependence on PRG-1 implicates piRNAs involvement in the mounting of a 22G response.

(5) Importantly, it is not the mll-1/B0250.8 transcript itself that was not shown to interact with WAGO-1 in the Seroussi et al. eLife paper (Lines 257-259). This study investigated sRNAs associated with every AGO, and computationally inferred the targets of each AGO using those enriched sRNA sequences. Therefore, it is the siRNAs antisense to mll-1/B0250.8 that were detected in association with WAGO-1, making it likely that WAGO-1 is the secondary AGO that targets this transcript. The argument the authors make holds true, but the authors should revise how they describe the evidence supporting that argument to accurately reflect the existing data.

Thank you for catching this mistake. We have updated the text to accurately reflect the results from the Seroussi et al 2023 publication:

“Recent work has shown that the N2 tmrl-1 transcript-derived small RNAs co-immunoprecipitated with WAGO-1, providing additional evidence that this transcript is regulated by the endogenous RNAi machinery”

(6) It seems likely that the authors explored the possibility that another antidote may be present in the third clade. Could they discuss what they did to rule out this explanation in lieu of piRNA/siRNA regulation?

We did not look for another antidote in the third clade because this clade is defined by the presence of an antidote and the absence of a toxin. Figure 3C shows the result of a cross between a third clade strain (NIC195) and XZ1516. The conclusion we draw from this experiment is that the antidote present in NIC195 provides near complete resistance to the XZ1516 toxin.

(7) Line 156, legend of Figure S3, and line 273: There was no marker used to indicate that these are the primordial germ cells. Best practices would indicate using a fluorescent marker (e.g., PIE-1 GFP or PGL-1 GFP or PRG-1 GFP, etc.) to definitively identify these as PGCs.

We agree with the reviewer’s point. As we do not have the perfect experiment, we do not definitively state that tmrl-1 transcripts localize in the primordial germ cells.

Minor comments:

(1) A minor suggestion: incorporating some of the results now shown in the supplementary figures - Figures S1, S3, and S4 - into the main figures may make the manuscript easier to read.

We constructed the manuscript in a way we thought was straightforward. The figures listed by the reviewer are supplemental to the main conclusions of the manuscript, so we decided to leave them as supplemental figures.

(2) Line 87, Figure S1A: include numbers in the y-axis.

The numbers are included on the y-axis and we explain the x-axis tick marks in the figure caption.

(3) Figures 1B, 2B, 3C, 4B, S1B, S4: statistical analyses missing.

We have added a summary of the statistical analysis to the captions of Figures 1B, 2B, 3C, and S1B. We added more detail from the analysis of 4A, which is the figure we draw conclusions from. Figure S4 is observational data, and the only conclusion drawn from that figure is that the N2 tmrl-1 gene encodes a toxin. It is toxic in 100% of individuals we looked at and therefore doesn’t warrant statistics.

(4) Line 100, "The rod progeny were all homozygous for QX1211 alleles at the locus on the right arm of chromosome V that displayed the allele frequency distortion in the mapping populations". Is this supported by data? While there is strong evidence to suggest it, the way it is currently written makes it seem that the rod progeny have been genotyped (by sequencing or PCR?). Is this the case? If not, the authors should revise the statement accordingly.

Yes, this is indeed the case and we have updated the text to reflect that we performed PCR of a QX1211-specific indel to verify the genotypes on the right arm of chromosome V.

(5) Figure 2A: lower panel missing x axis label.

The top panel is a cartoon representation of a NILs, and the x axis is labeled for the top panel, highlighting the mapped element.

(6) Line 140 to 148: The authors should provide data to support these statements.

Realizing i skipped this one – these are the lines they are referring to -> Long-read RNA sequencing revealed two distinct mll-1 isoforms, a short isoform with three predicted exons and a long isoform with eight predicted exons (Fig. S2A). We constructed plasmids with inducible versions of each mll-1 isoform. When we injected susceptible strains with the short mll-1 isoform array, every F1 individual carrying the array died, with 64% of larvae exhibiting the rod phenotype, indicating that uninduced expression levels of the short mll-1 isoform are sufficient to induce lethality. By contrast, we were able to isolate susceptible strains that maintained the long mll-1 isoform array or a short mll-1 isoform array with a premature stop codon in mll-1. We observed no rod progeny upon induction of these arrays, indicating that the short isoform encodes the functional toxin, and that the toxin acts as a protein.

(7) Line 193: It would be interesting to see if there is structural conservation between mll-1 and B0250.8 using alpha-fold. Have the authors done this?

We did attempt to look for structural conservation but we found the confidence in the structural predictions to be very low, which didn’t warrant a comparison.

(8) Line 206-207: Could the authors explain why the frequency of the rod phenotype is so low when presumably over-expressing B0250.8? Does this indicate that B0250.8 is not as functional a toxin as mll-1, or is it sufficiently repressed by sRNAs and not actually overexpressed? Further, what are "abnormal" phenotypes? This should be clarified for the reader.

It is likely that the overexpression and misexpression of toxic proteins is causing the abnormal phenotypes. The rod phenotype probably manifests when the gene is expressed at the appropriate developmental stage and tissue to cause the phenotype, whereas abnormal phenotypes manifest when the expression is not in the correct stage or location. A summary of the observed phenotypes is provided in Supplementary Table 7.

(9) Line 216 and thereafter: indicate that B0250.8 is now referred to as mll-1.

We incorporated this suggestion.

(10) Line 228-231: missing to state that this is shown in Figures 4A-B.

This and the following comment suggests that we did not provide enough clarity in this section. We modified the line to the following:

Consistent with this report, in an agar plate-based preliminary assay we observed that ~15% of ∆mut-16 progeny arrest at various larval stages, and 2% of progeny are rod, which is suggestive of derepression of tmrl-1 in N2.

This lets readers know that this initial characterization of the mut-16 knockout strain is different from the data presented in figure 4.

(11) Line 230: the Figure shows ~25% of arrest for the deletion mutant of mut-16, but the text says ~15%.

The 15% the reviewer points out was obtained from a preliminary agar plate-based experiment where we attempted to characterize the mut-16 deletion strains. We turned to a more high-throughput approach to screen through more animals for each genotype, which we report in figure 4.

(12) Line 233: TOF, and not animal length, was compared. The authors should indicate that TOF is used as a proxy for animal length.

We made the suggested change. The new sentences read:

To do so, we compared time of flight (TOF) measurements—a proxy for animal length, developmental stage, and growth rate (28)—between a strain with a single knockout of mut-16 and one with a double knockout of mut-16 and the N2 tmrl-1 (a strain with a single knockout of the N2 tmrl-1 served as a negative control). We observed a reduction in TOF and an increase in the fraction of worms in larval stages in the mut-16 knockout strain, and these effects were partially rescued in the double knockout strain (Fig. 4).

(13) Line 237-239: This claim may be overstated without additional data. Consider adding a "likely" to the statement.

The line in question:

These results indicate that the reduced growth rate observed in the mut-16 knockout strain is partially mediated by derepression of the N2 mll-1 allele.

We modified it to reflect the reviewer’s concern:

These results indicate that the reduced growth rate observed in the mut-16 knockout strain is partially mediated by the presence of the N2 tmrl-1 allele, likely because tmrl-1 is derepressed in mut-16 knockout strains.

(14) Line 257: Figure S5C should be moved to line 259.

We made the suggested move.

(15) Is the name mll-1 firmly established? We ask because MLL1 is a human mutation commonly associated with leukemia, and it may lead to some confusion in the field. This is a minor point, but we wanted to bring it forth.

This name was not firmly established. We modified the names to not overlap with known gene names:

tmrl-1 - Toxin-induced Maternal Rod Lethality

amrl-1 - Antidote of Maternal Rod Lethality
